# Investigation of the Fabrication of Diamond/SiC Composites Using α-Si_3_N_4_/Si Infiltration

**DOI:** 10.3390/ma16186252

**Published:** 2023-09-17

**Authors:** Bo Xing, Yingfan Zhang, Jinzhui Zhao, Jianyu Wang, Guoqin Huang

**Affiliations:** 1 Institute of Manufacturing Engineering, Huaqiao University, Xiamen 361021, China; xb@zzsm.com (B.X.);; 2 State Key Laboratory for High Performance Tools, Zhengzhou Abrasive Grinding Research Institute Co., Ltd., Zhengzhou 450001, China

**Keywords:** diamond/SiC composites, optimization model, silicidation, antiadhesion

## Abstract

Diamond/SiC (Dia/SiC) composites possess excellent properties, such as high thermal conductivity and low thermal expansion coefficient. In addition, they are suitable as electronic packaging materials. This study mainly optimized the diamond particle size packing and liquid-phase silicon infiltration processes and investigated a method to prevent the adhesion of the product to molten silicon. Based on the Dinger–Funk particle stacking theory, a multiscale diamond ratio optimization model was established, and the volume ratio of diamond particles with sizes of D20, D50, and D90 was optimized as 1:3:6. The method of pressureless silicon infiltration and the formulas of the composites were investigated. The influences of bedding powder on phase composition and microstructure were studied using X-ray diffraction and scanning electron microscopy, and the optimal parameters were obtained. The porosity of the preform was controlled by regulating the feeding amount through constant volume molding. Dia/SiC-8 exhibited the highest density of 2.73 g/cm^3^ and the lowest porosity of 0.6%. To avoid adhesion between the sample and buried powder with the bedding silicon powder, a mixed powder of α-Si_3_N_4_ and silicon was used as the buried powder and the related mechanisms of action were discussed.

## 1. Introduction

The development of electronic equipment for high power density and integration presents higher requirements for the performance of electronic packaging materials [1,2]. Heat sinks are a class of materials that can transfer heat efficiently, consume large amounts of thermal energy, and are widely used in heat dissipation applications [3]. In high heat flow situations, more emphasis is placed on heat dissipation efficiency. Currently, the most ideal heat sink material is single-crystal diamond, but it is difficult to manufacture, limited in size, and costly. Therefore, other diamond composites are used, such as diamond/copper and diamond/aluminum [4,5]. However, the presence of thermal shocks may cause the diamond/metal interface to separate and thus reduce the heat dissipation effect. Considering the high theoretical thermal conductivity of diamond and silicon carbide, diamond/silicon carbide (Dia/SiC) composites represent one of the materials with development potential. Dia/SiC composites, which are easier to prepare than large diamond substrates [6], possess a wide range of application prospects given their high thermal conductivity, low coefficient of expansion, low density, and high strength [7,8].

The existing methods for preparing Dia/SiC composites include high-temperature and high-pressure (HTHP) sintering [9,10], precursor conversion [11], vacuum discharge plasma sintering [12], and hot isostatic pressing [8,13]. The application of hot isostatic pressing and HTHP sintering methods to prepare composite materials is limited by their expensive equipment [14]. The precursor conversion method is characterized by a long preparation period and unevenly distributed pores in the product. The infiltration methods include pressure [15,16] and pressureless infiltration [17,18]. In the pressureless infiltration method, the melt spontaneously penetrates the preform under capillary pressure without an external force and undergoes a complex chemical reaction with it to form a dense ceramic composite material [19,20]. This method is an extremely competitive production process characterized by simple equipment requirements, low preparation costs, a short production cycle, high product density, and a lack of deformation during sintering. Herrmami et al. prepared a silicon-bonded diamond material using the pressureless infiltration method; the wear resistance of the material was significantly improved using hard metals and ceramics [21]. Zhang et al. investigated the interface region and formation mechanism of Dia/SiC composites prepared with infiltration [22]. The results showed that the composite contained three main phases: diamond, SiC, and residual Si. Zheng et al. prepared Dia/SiC composites using silicon vapor reactive infiltration [17]. In the aforementioned study, the vacuum conditions slowed down the graphitization of diamond, and solid interface bonding was formed between diamond and SiC within the range of 1550–1650 °C.

Although the pressureless infiltration process is useful for the successful preparation of Dia/SiC composites, certain key problems are encountered during its use. Dia/SiC composites prepared using pressureless infiltration have the disadvantage of an uneven diamond distribution. In the reinforcing phase of Dia/SiC composites, diamond content and size significantly affect the preparation process and product performance in terms of the fluidity of the preformed slurry, formation of a diamond thermal network, densification of the composite, etc. Multiscale filler compounding can be employed to adjust the fluidity of a ceramic slurry and improve the physical properties of ceramic products [23]. Following the maximum packing theory, Zhu et al. prepared concrete with excellent performance [24]. Mao established a filling model to guide the compounding of multiscale, thermally conductive particles [25]. Dia/SiC composites were prepared using mixed-particle-size diamonds. On the one hand, mixed-particle-size diamonds are beneficial for the densification of composite materials. On the other hand, the thermal conductivity of the composite material is also improved.

Typically, pure silicon powder is used as bedding powder when Dia/SiC composites are prepared with the pressureless siliconizing method. Elemental silicon is melted and evaporated at a high temperature to provide a silicon source for the porous preform [16]. However, the melting point of elemental silicon is 1410 °C [26]. The liquid silicon that does not participate in the reaction wraps the sample within the temperature range of 1500–1700 °C, and it is difficult to separate the product from the remaining silicon after cooling. To solve the problem of adhesion between the product and molten silicon, a mixture of α-Si_3_N_4_ and silicon powder is used as a silicon source. α-Si_3_N_4_ is used as a filter screen to reduce the adhesion of silicon powder to the product [27,28]. In addition, α-Si_3_N_4_ is unstable above 1300 °C and can react with carbon to form silicon carbide [29].

In this study, the mixed proportion of diamond particles with three different sizes was designed based on the Dinger–Funk equation of the maximum packing theory. The influence of the mixture of α-Si_3_N_4_ and silicon powder on phase composition and product adhesion status was investigated using X-ray diffraction and scanning electron microscopy (SEM). Finally, the optimal parameters were obtained. The highest density and lowest porosity values obtained were 2.73 g/cm^3^ and 0.6%, respectively. The use of a mixture of α-Si_3_N_4_ and silicon as bedding powder generates silicon carbide at high temperatures and effectively avoids adhesion of the product to molten silicon.

## 2. Optimization of Diamond Particle Gradation for Diamond/SiC Composites

**Figure 1:**
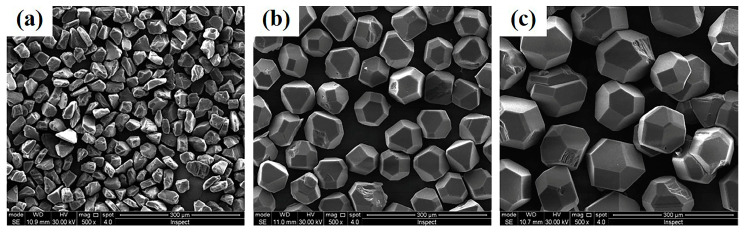
Morphology of diamonds particles with sizes of: (**a**) D20, (**b**) D50, and (**c**) D90.

In this work, three particle sizes of diamond were used, including D20, D50, and D90, with corresponding average particle sizes of 20, 50, and 90 µm, respectively, as shown in Figure 1a–c. Diamonds with an average grain size of 90 µm and 50 µm are mainly dodecahedral in shape with a more complete crystal form. Diamonds with an average grain size of 20 µm are mainly obtained from the crushing of large diamond grains. Their shape appears as angular, oval, with less sheets and needles present. Therefore, for calculation convenience, diamond particles with different shapes were uniformly simplified as sphere. Based on the Dinger–Funk equation [24] of the maximum packing theory, the mix proportion was designed as follows. The ideal model of the multiparticle diamond distribution is shown in Equation (1).

(1) $$U(D_{p})=\frac{D_{p}^{n}-D_{\min}^{n}}{D_{\max}^{n}-D_{\min}^{n}}$$

where *U(D_p_)* is the cumulative percentage of the filler with a particle size less than *D_p_*; *D_p_* is the particle size of the filler in µm; *D_min_* and *D_max_* are the minimum and maximum diameters of the filler in the system in µm, respectively; and *n* is the distribution modulus (0.37 in the densest accumulation).

When the accumulated particle-size distribution of the filler was close to the Dinger–Funk distribution after compounding the multiscale diamond, the gap of the large particle filler in the composite material was matched with the particle size of the small particles. For the multiscale filler compounding system, the cumulative distribution satisfied Equation (2).

(2) $$U^{\prime }(D_{p})=\sum_{i=1}^{m}a_{i}U_{Di}$$

where *U′*(*D_p_*) is the cumulative percentage of the “particle size less than *D_p_*” filler; *a_i_* is the proportion of the *i*th particle size filler to the total filler volume fraction; *i* = 1, 2, 3…*m* (*m* is a positive integer); and *U_Di_* is the cumulative distribution function of the *i*th particle size filler.

To achieve a filler particle size accumulation distribution *U′*(*D_p_*) close to the filler particle size accumulation distribution *U*(*D_p_*) in the ideal model, the minimum sum of the squared differences between the distribution and objective functions of the perfect model was constrained using the least-squares method. The constructed model should satisfy Equation (3).

(3) $$\left\{\begin{array}{l} \min\int_{D_{\min}}^{D_{\max}}{\left(U^{\prime }(D_{p})-U(D_{p})\right)}^{2}dD=\min\int_{D_{\min}}^{D_{\max}}{\left(\sum_{i=1}^{m}a_{i}U_{Di}-\frac{D_{p}^{n}-D_{\min}^{n}}{D_{\max}^{n}-D_{\min}^{n}}\right)}^{2}dD\\ a_{1}+a_{2}+a_{3}+\cdot \cdot \cdot +a_{i}+\cdot \cdot \cdot +a_{m}=1\\ 0\leq U_{D1},U_{D2},U_{D3},\cdot \cdot \cdot ,U_{Di},\cdot \cdot \cdot ,U_{Dm}\leq 1 \end{array}\right.$$

**Figure 2:**
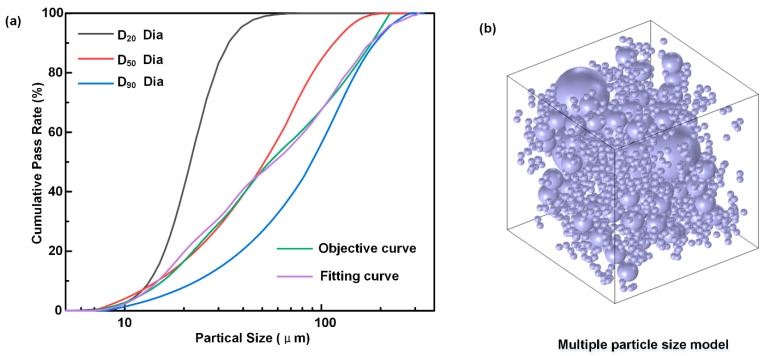
Cumulative distribution curve and optimization model: (**a**) cumulative distribution curves of diamonds with different sizes, (**b**) multiscale diamond matching optimization model.

For Equation (3), the optimal volume fraction share of each component of the multiscale filler, *a*_1_, *a*_2_, and *a*_3_, can be obtained after simple programming using Matlab’s nonlinear programming function with the cumulative distribution of each component of the filler as a covariate. Here, three diamond sizes were selected, and the cumulative particle-size distribution is shown in Figure 2. The cumulative distribution of diamonds with three particle sizes is shown in the cumulative distribution curve, and an ideal model of the cumulative distribution after compounding is presented. Figure 2a shows that the cumulative distribution of each diamond typically presents an “S” curve, which significantly differs from the curve of the Dinger–Funk ideal distribution model. However, it was very close to the ideal distribution curve after model optimization. Figure 2b shows the multiscale diamond matching optimization model. According to the optimization results, the preferred volume ratio of diamonds D20, D50, and D90 is 1:3:6.

## 3. Experimental Setup

### 3.1. Materials

Diamonds with a mixture of D20, D50, and D90 at a volume ratio of 1:3:6 were purchased from ZhongNan Diamond Co., Ltd., Nanyang, China, Silicon (purity 99.9%) was obtained from Beijing October New Material Technology Co., Ltd., Beijing, China, Graphite (purity 99.9%) was provided by Qingdao Tianyuanda Technology Co., Ltd., Qingdao, China, Silicon and graphite were the main raw materials for the preparation of silicon carbide. Generally, it is easier to generate silicon carbide using Si powder and graphite with fine particle sizes. However, the nanopowder easily oxidizes and agglomerates. The higher the content of Si and carbon powder, the more silicon carbide is generated, which can affect the performance of the composite. Thus, the silicon and graphite powders used in this work had an average grain size of 5 µm. Silicon nitride (α-Si_3_N_4_) was supplied by Qinhuangdao Yinuo Technology Co., Ltd., Qinhuangdao, China. A phenolic resin provided by Shandong Shengquan New Material Co., Ltd., Jinan, China was used to bond the diamond, graphite, and silicon powder. Phenolic resin was carbonized and cracked to form activated carbon, which can react with silicon to form silicon carbide. Although diamond can react with Si powder to form silicon carbide at high temperatures, the penetration depth of silicon powder is limited; therefore, additional graphite powder was required. Diamond, graphite, silicon powder, and phenolic resin with a volume ratio of 40:20:20:20 were mixed to prepare the Dia/SiC composite material.

### 3.2. Preparation of Dia/SiC Composites

**Figure 3:**
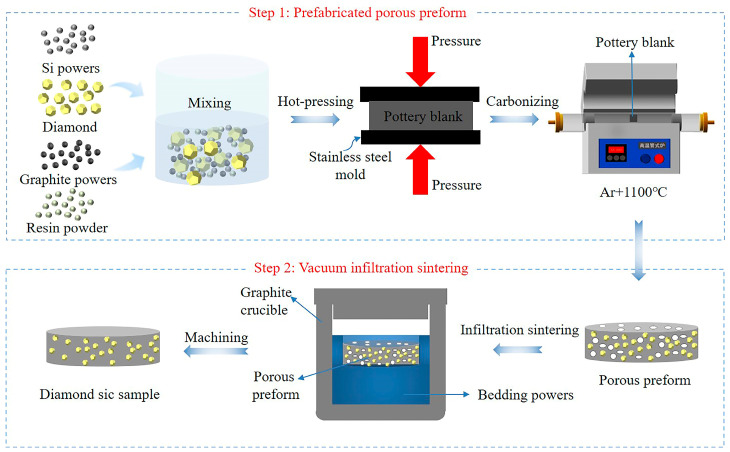
Schematic diagram of the preparation of Dia/SiC composites.

**Figure 4:**
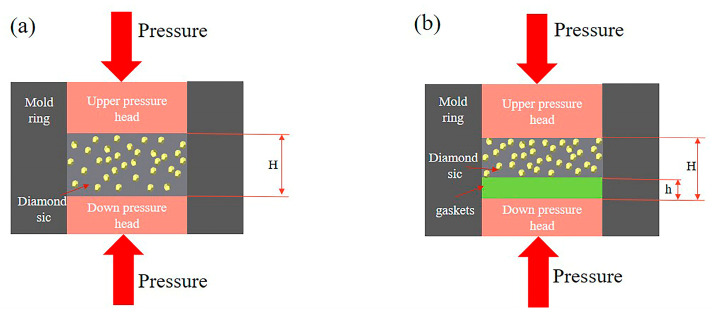
Schematic diagram of using gaskets to control reduction ratio of Dia/SiC composites: (**a**) without gaskets when pressing samples, (**b**) adding gaskets when pressing samples.

The composite was prepared using the vacuum infiltration process. The vacuum sintering furnace (ZT-40-21Y) used was purchased from Shanghai Chenhua Technology Co., Ltd., Shanghai, China. The highest sintering temperature of the sample was 1700 °C at a vacuum degree of 0.01 Pa. The steps of Dia/SiC composite preparation are illustrated in Figure 3. First, the pottery blank was pressed using the constant volume method at 100 °C and 50 kN. Gaskets are used to determine the reduction ratio for controlling the density and porosity of Dia/SiC, as shown in Figure 4. Figure 4a is a schematic diagram of pressing samples with fixed molds without gaskets, while Figure 4b is a schematic diagram of pressing samples with added gaskets. In Figure 4, H is the height dimension of the fixed mold height, and h is the thickness dimension of the added gasket. Here, h/H ratios of 0, 4%, 6%, 8%, and 10% are labeled as Dia/SiC-0, Dia/SiC-4, Dia/SiC-6, Dia/SiC-8, and Dia/SiC-10, respectively.

Thereafter, the ceramic blank was carbonized in an argon furnace at 1100 °C to obtain a preform with a certain strength. The preform was buried in the bedding powder, which contained a large amount of silicon (Figure 3). The elemental silicon was melted and formed silicon vapor at a high temperature, which could be used to sinter the sample. Notably, the silicon content of the buried powder was considerably greater than the chemical equivalent required to sinter the sample. The graphite crucible containing the sample and bedding powder was placed in a high-temperature vacuum furnace. Finally, a robust product was obtained using low-pressure siliconizing at 1500 °C, 1550 °C, 1600 °C, 1650 °C, and 1700 °C with the reaction of silicon and carbon to form SiC.

### 3.3. Characterization

A Field-emission SEM (FE-SEM; Apreo2, Thermo Fisher, Waltham, MA, USA) was employed to inspect diamonds and composites. The microstructure and the phase composition in the composite were analyzed with energy-dispersive X-ray spectroscopy (EDS). Here, XRD (D8 Advance, Bruker, Billerica, MA, USA) was performed using Cu-Kɑ radiation (λ = 0.154 nm) at ambient temperature within a scanning range of 10–60°.

## 4. Results and Discussion

### 4.1. Mechanism of Dia/SiC Sample Formation

**Figure 5:**
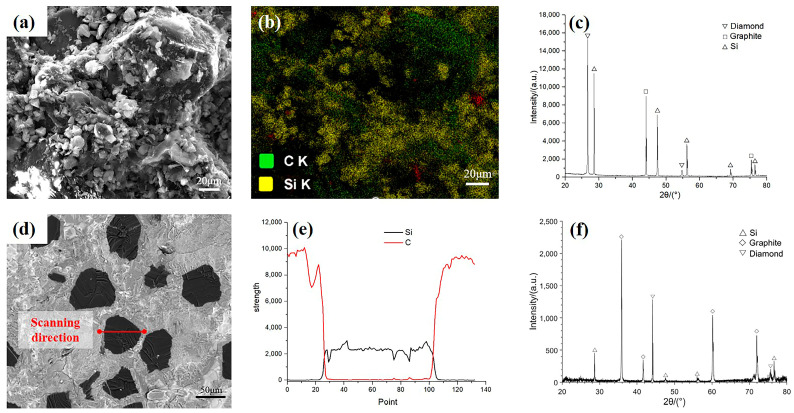
SEM, EDS and XRD images of the preform and sample: (**a**–**c**) SEM image, EDS image, XRD image of the fracture of preform, (**d**–**f**) SEM image, EDS image, XRD image of the fracture of sample.

Figure 5 shows the SEM images, EDS spectra, and XRD patterns of the fracture surfaces of the preforms and products. Figure 5a–c show the cross-section of the preform. The diamond was in the skeleton phase, as the size of the diamond grits was larger than that of other materials. The diamond surface was coated with graphite and silicon powder; therefore, the diamond particles covered with other powder materials were spherical and dispersed. No evident particle agglomeration was observed, and the diamond particles were tightly packed without large gaps, indicating that the inner microstructure of the preform was tense after carbonization. In addition, XRD revealed that diamond, graphite, and silicon were present in the preform since amorphous carbon does not have an XRD signal. This showed that no chemical reaction occurred between diamond and silicon at 1100 ℃, and the carbonization of the phenolic resin was relatively sufficient, as shown by the EDS results. Most of the graphite in the preform originated from the raw graphite material. The rest probably originated from the diamond, which was slightly graphitized in the argon protective atmosphere, and there was no influence on the composition. Figure 5d–f show the cross-section of the product. The SEM image shows that the product was dense and that the diamond was embedded in the matrix. No gap was observed in the interface between the diamond and matrix. The EDS according to the scanning direction in Figure 5d, the spectra of the diamond in the matrix showed that silicon gradually increased and carbon decreased, indicating that elemental diffusion occurred between the diamond and the matrix and that a new substance may have formed. The XRD patterns revealed the presence of diamond, silicon carbide, and elemental silicon in the product. This indicated that the silicon was in excess and that the graphite completely reacted with the silicon to form silicon carbide during high-temperature sintering. The matrix contained silicon carbide and elemental silicon. These results confirmed the successful preparation of Dia/SiC composites.

### 4.2. Influence of the Pressing Process on the Density and Porosity of the Dia/SiC Samples

**Figure 6:**
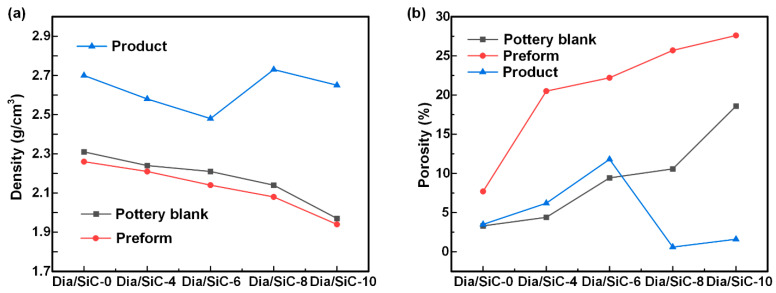
Variations in sample density (**a**) and porosity (**b**) noted with the hot pressing process.

To investigate the influence of the pottery blank pressing process on the product performance, the density and porosity of different Diamond/SiC composite samples were tested. Here, the ceramic pottery blank was prepared using constant volume molding because the siliconizing process required the preform to be porous. Here, the porosity of the preform was controlled by regulating the feeding amount, thereby regulating the product density. For example, the feeding amount of Dia/SiC-10 was 10 wt.% less than that of Dia/SiC-0. The test results obtained for Dia/SiC-0, Dia/SiC-4, Dia/SiC-6, Dia/SiC-8, and Dia/SiC-10 are shown in Figure 6. Figure 6a shows that the density of the pottery blank decreased as the feeding amount decreased. Using the same formula and process, the sample density exhibited the following order: product > pottery blank > preform. The densities of the preform and blank were extremely close. Considering that the product was prepared by vacuum siliconizing the preform, the product density exceeded that of the preform. The lower density of the preform compared with that of the pottery blank was due to the mass loss caused by the carbonization of the phenolic resin. The preform was obtained by carbonizing the corresponding pottery blank; therefore, their density change trends were the same. The product density varied because the porosity and pore size of the preform significantly influenced the vacuum siliconizing. Dia/SiC-8 exhibited the highest density of 2.73 g/cm^3^. Figure 6b shows that the porosity variation of different samples was similar to the density variation. The porosity of the pottery blank and preform increased as the feeding amount increased, and the porosity of the preform exceeded that of the pottery blank. The product consistently exhibited lower porosity, and Dia/SiC-8 exhibited the lowest porosity of 0.6%.

### 4.3. Influence of Bedding Powder Types on Dia/SiC Sample Preparation

**Figure 7:**
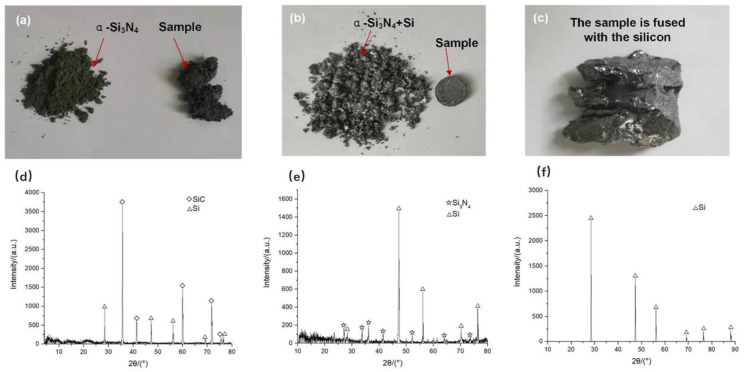
State of samples prepared using different bedding powders and the composition of bedding powders after sintering: (**a**) sample prepared with α-Si_3_N_4_ as bedding powder, (**b**) sample prepared with α-Si_3_N_4_ and Si as bedding powder, (**c**) sample prepared with pure Si as bedding powder and (**d**–**f**) the composition of the corresponding bedding powder shown (**a**–**c**).

To investigate the influence of bedding powder types on the sample preparation, the state of the samples prepared with different bedding powders and the composition of the bedding powders were analyzed after sintering. Figure 7 shows the samples prepared using α-Si_3_N_4_ powder, a mixed powder of α-Si_3_N_4_ and Si, and pure Si powder as bedding powder, as well as the state of the bedding powder after sintering. As shown in Figure 7a–c, when pure α-Si_3_N_4_ was used as the bedding powder, the obtained product exhibited a loose structure without strength, and the rest of the α-Si_3_N_4_ remained in powder form after sintering. When pure Si powder was used, the sample and molten silicon were severely adhered and were difficult to separate after sintering. When the mixed powder of Si and α-Si_3_N_4_ was used, the sample was completely formed and could be easily separated from the bedding powder after sintering. The remainder of the bedding powder formed a type of corporation structure under the effect of the melted silicon. As shown in Figure 7d–f, only SiC and diamond remained in the bedding powder after sintering using α-Si_3_N_4_ as the bedding powder. Diamond is derived from preforms, whereas SiC is mainly formed through the reaction of α-Si_3_N_4_ and graphite. Thus, α-Si_3_N_4_ can be used as a silicon source to generate silicon carbide at a high temperature. Therefore, the mixed powder of α-Si_3_N_4_ and Si can be used to effectively avoid the adhesion of the product to molten silicon. The optimal amount of silicon powder in the mixed powder was 60–70%.

### 4.4. Change Trend in Buried Powder Composition with the Increase of Sintering Temperature during Vacuum Infiltration

**Figure 8:**
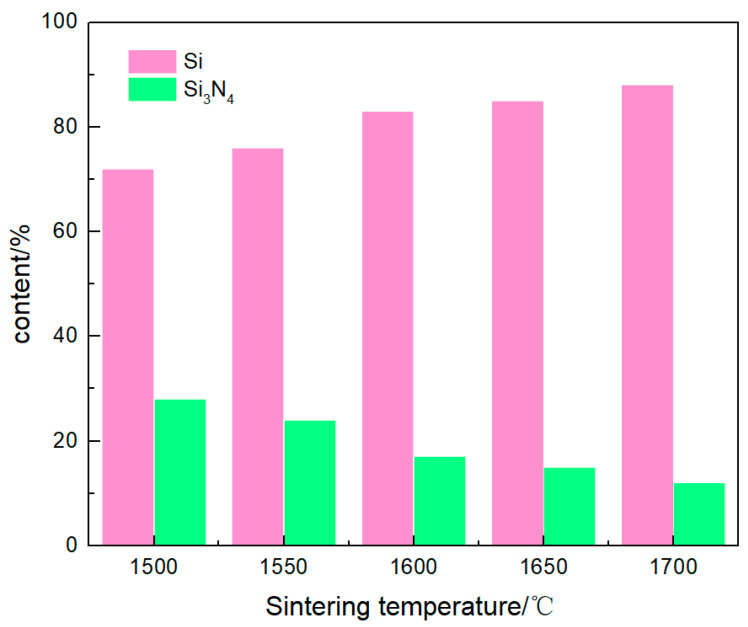
Change trend of Si and Si_3_N_4_ compositions in buried powder versus the sintering temperature of vacuum infiltration.

Figure 8 shows the change trend of Si and Si_3_N_4_ compositions in the buried powder versus the sintering temperature of the vacuum infiltration. As the sintering temperature increased, the decomposition degree of silicon nitride became more sufficient. The silicon nitride content gradually decreased, and the silicon content gradually increased correspondingly. When the temperature increased from 1500 °C to 1700 °C, the silicon content increased from 72% to 88%, representing a 22.2% increase. In contrast, the silicon nitride content decreased from 28% to 12%, representing a 57.1% decrease. The amount of silicon nitride decomposition increased, and the decomposed silicon preferentially participated in the osmotic chemical reaction. Although the free silicon evaporated and participated in the osmotic reaction, the amount was relatively limited, and the proportion of free silicon gradually increased.

## 5. Conclusions

In summary, this study mainly optimized the diamond particle size packing and liquid-phase silicon infiltration processes and investigated a method to prevent the adhesion of the product to molten silicon, which provides an alternative solution for the design and preparation of Dia/SiC composites. The primary conclusions are listed as follows:

(1) A multiscale diamond ratio optimization model based on the Dinger–Funk particle stacking theory was established, and the optimal volume ratio for diamonds with sizes of 20, 50, and 90 µm was 1:3:6.
(2) Dia/SiC composites were prepared using the low-pressure siliconizing method at 1600 °C, and the effect of the preparation process on product density was investigated. The highest density and lowest porosity obtained were 2.73 g/cm^3^ and 0.6%, respectively.
(3) A mixed powder of α-Si_3_N_4_ and Si was used as the bedding powder to effectively avoid the adhesion of the product to molten silicon. α-Si_3_N_4_ was used as a silicon source to generate silicon carbide at high temperatures.

## Data Availability

Data available on request.
